# Direct Percutaneous Mitral Annuloplasty in Patients With Functional Mitral Regurgitation: When and How

**DOI:** 10.3389/fcvm.2019.00152

**Published:** 2019-11-06

**Authors:** Tomasz Gasior, Mara Gavazzoni, Maurizio Taramasso, Michel Zuber, Francesco Maisano

**Affiliations:** ^1^University Heart Center, University Hospital Zurich, Zurich, Switzerland; ^2^Division of Cardiology and Structural Heart Diseases, Medical University of Silesia, Katowice, Poland

**Keywords:** direct percutaneous annuloplasty, mitral annuloplasty, mitral regurgitation, transcatheter valve repair, mitral valve interventions, mitral valve imaging, patient selection, mitral annulus

## Abstract

Mitral regurgitation (MR) is a frequent valvular disease among patients deemed too high risk for surgery. Echocardiography along with CT is the primary diagnostic tool for MR and offers a comprehensive 3D assessment in patient selection and screening for the optimal treatment method. The direct percutaneous mitral annuloplasty addresses the underlying mechanisms of functional MR with a less invasive, catheter-based approach. The here-described techniques proved a sufficient safety profile, delivered significant MR reduction in most of the cases, and were associated with a notable improvement of symptoms. Although long-term outcome assessment is needed to support these early reports, the percutaneous mitral annuloplasty is likely to set a new standard of treatment in the forthcoming future.

## Introduction

Mitral regurgitation (MR) is the most frequent valve disease in developed countries (1). Referred to as secondary or as functional mitral regurgitation (FMR), it is caused by sustained left ventricle injury in the course of myocardial infarction or certain forms of cardiomyopathy, leading to left ventricle remodeling. That may result in the displacement of the papillary muscles and mitral annulus (MA) dilatation, yet the leaflet structure remains usually intact (2). Patients presenting FMR procure an unfavorable prognosis, resulting in over two-fold higher mortality when compared to primary MR and extremely high risk of heart failure (HF rate at 5 years, 78%) (3, 4).

Building on the years of experience with mitral valve replacement and repair procedures, together with a desire for a less invasive approach, multiple percutaneous technologies have emerged as a feasible and convenient therapeutic option for patients with MR. They can be classified depending on the anatomical and pathophysiological grounds: the indirect and direct annuloplasty, left ventricle (LV) remodeling devices, and leaflet and chordal repair procedures. This review aims to summarize current principles for patient selection and pre-procedural planning for direct transcatheter annuloplasty followed by the procedural know-how.

## Mitral Valve Annulus Structure and Function

**Figure 1:**
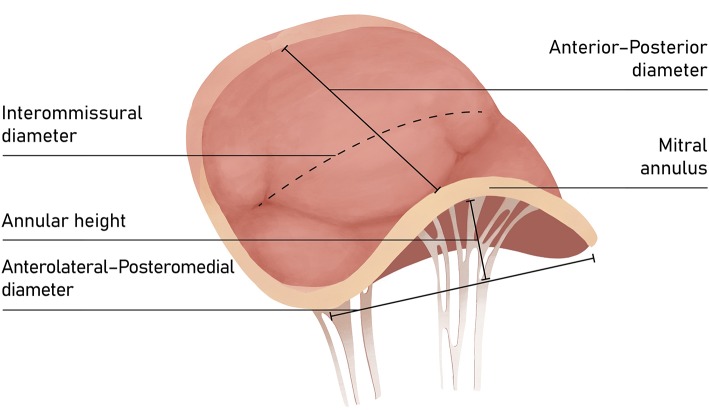
The saddle-shape configuration of the mitral valve. The nonplanar shape is believed to significantly reduce the mechanical strains on the posterior leaflet during systole and optimize force distribution among mitral apparatus.

**Figure 2:**
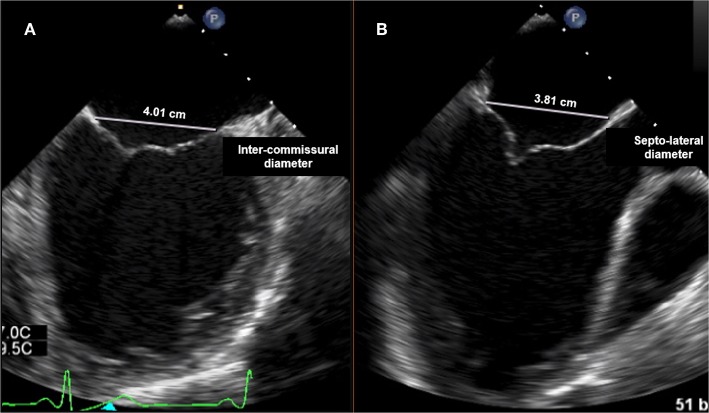
Transesophageal echocardiography (TTE): **(A)** intercommissural view focused on MV for intercommissural diameter measurement; **(B)** mid-esophageal Long-axis view (ME LAX View) for septo-lateral (antero-posterior) annulus measurement.

The mitral valve complex consists of valvular (annulus, commissures, leaflets) and tension (papillary muscles, chordae tendineae) components. MA is the functional component of the valve characterized by a non-planar, saddle-shape frame, believed to be involved in reducing stress on the valve elements during systole (Figure 1) (4). For interventional purposes, the annulus is regarded as the area of the attachment of the valve leaflets to the atrial part of the surrounding heart tissue. The anterior part consists of 1/3 of the valve circumference and incorporates a fibrous thick tissue, supported at each side of the base of the leaflet by the left (anterolateral) and right posteromedial fibrous trigones (5). Of more importance, the MA's three-dimensional geometry varies through the cardiac cycle. The standard correlation between the septolateral (antero-posterior) and transverse (inter-commissural) diameters of the MA is measured during systole and typically comes close to 3:4 (Figure 2). This can differ among patients with chronic MR, specifically when the leaflet coaptation is lost, even in the non-prolapsing segments (6).

Comprehensive assessment of MA still pose a challenge since different methods and settings have been used to establish the cutoff value. Since the 3D shape of MV annulus is other than simple, only a thorough examination is believed to be sufficient in clinical practice. The current cutoff for annular dilatation is based on transesophageal measurements of the end-diastolic antero-posterior MA diameter derived from a small group of 49 patients referred to MV surgery. This value is regarded as 35 mm and is still the most widely used in intraoperative setting (7).

In the non-surgical setting, the largest population-based echocardiographic dataset available to date is that of Dwivedi et al. This study was conceived specifically to determine standard mitral and tricuspid annulus dimensions with the use of 2D TTE. Gender-specific mean diameters of MA were 3.44 cm in males and 3.11 cm in females at end-systole and 3.15 cm in males and 2.83 cm in females at end-diastole. Interestingly, MA reduction in systolic phase was found to be up to 25% (8).

**Figure 3:**
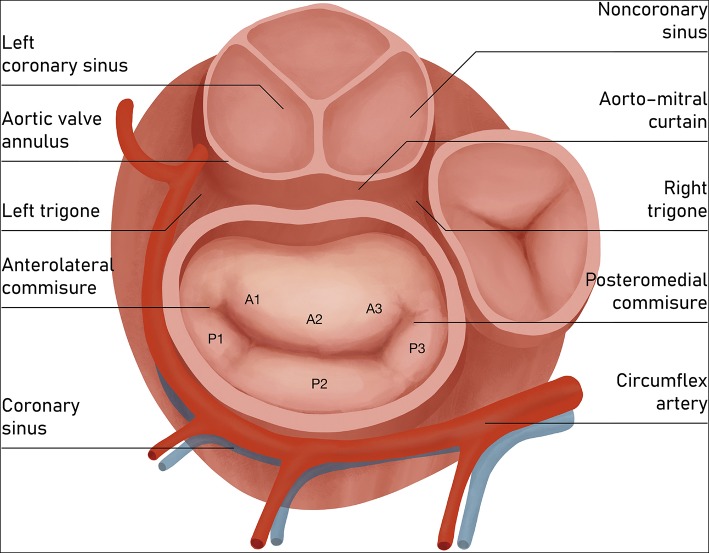
Atrial view of mitral valve. Components of mitral valve apparatus and the adjacent structures. The posterior leaflet of the mitral valve composes ~3/5 of the annular circumference and comprises three individual scallops identified as P1 (anterior or medial scallop), P2 (middle scallop), and P3 (posterior or lateral scallop). The three corresponding segments of the anterior leaflet are A1 (anterior segment), A2 (middle segment), and A3 (posterior segment).

Some pivotal structures are closely located to the MA and should always be thoughtfully considered during all annuloplasty procedures: (a) the circumflex artery, which runs parallel to MV plane; (b) the coronary sinus, which lies roughly around the base of the posterior leaflet; (c) the bundle of His, situated close to posteromedial commissure; and (d) the non-coronary and left coronary aortic cusps, located adjacent to the base of the anterior leaflet (Figure 3). The aorto-mitral curtain, composed mainly of muscular fibers, while separating aortic and mitral structures, serves as inch point during systolic phase for counterbalancing the mitral tethering forces (9).

## Annuloplasty Techniques

The implementation of annuloplasty technique in the mitral valve repair has been initially developed in the surgical setting to restore the normal annular shape and dimensions by correcting posterior annular dilatation in symptomatic FMR patients. A standard surgical treatment comprises of annular ring reduction, aiming to improve leaflet apposition, relieve tension on the leaflets by optimizing the coaptation zone, preserve leaflet mobility, and prevent further annular dilatation.

There are various surgical annuloplasty devices on the market, including flexible or semirigid rings. Ring sizing is performed following Carpentier's principles, while the intra-operative measurement involves the intercommissural diameter and the area of the anterior leaflet.

Transcatheter annuloplasty has been developed in the field of transcatheter MV repair to fill the therapeutic gap for high-risk surgery patients with FMR and to prove its position as a solid percutaneous alternative to the edge-to-edge treatment or an adjuvant strategy among the FMR patients. In the recently published study, apart from the improvement of mitral parameters and symptoms, the investigators demonstrated that the use of Cardioband (Edwards Lifesciences) might result in lower rehospitalization and mortality when compared to MitraClip treatment (Abbott), especially when low-LVEF patients are concerned (10).

**Figure 4:**
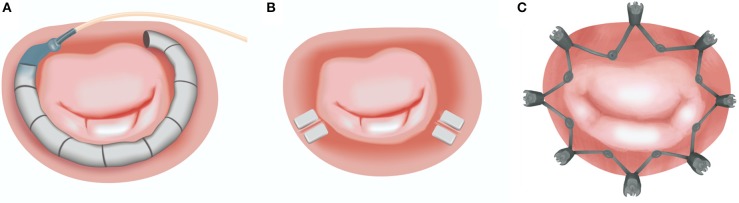
Currently available transcatheter direct annuloplasty systems: **(A)** Cardioband (Edwards Lifesciences); **(B)** Mitralign (Mitralign Inc.); **(C)** Millipede (Boston Scientific Corp.). Image Courtesy of Millipede, Inc. www.millipedemedical.com All rights reserved.

There are currently a number of devices under investigation for both indirect and direct percutaneous approach. Indirect techniques depend on the device introduction and placement within the MA through the parallel coronary sinus, whereas direct annuloplasty requires retrograde access to the left ventricle or trans-septal puncture. The latter techniques deliver the device within the close proximity of the MA (Table 1; Figure 4).

**Table 1:** Currently available transcatheter direct annuloplasty systems (transapical approach devices have not been listed).

	**Cardioband** **(Edwards Lifesciences)**	**Mitralign** **(Mitralign Inc.)**	**Millipede** **(Boston Scientific Corp.)**
Access	Transseptal	Retrograde	Transseptal
Position	Supra-annular	Cross-annular	Supra-annular
Repositionable	Yes	Yes	Yes
Clinical status	CE Mark	CE Mark	Investigational use

The method of direct transcatheter annuloplasty is a recognized therapy for inoperable MR patients, which offers a safer profile when compared to conventional surgery. Although it represents a technically complex procedural approach, it eliminates some of the anatomical restrictions of the indirect technique. These limitations comprise the variable distance between the coronary sinus and the annular plane, as well as the frequently close location of the circumflex artery exposed to iatrogenic injury. To date, there are two CE-approved direct annuloplasty devices, the Cardioband (Edwards Lifesciences) and the Mitralign (Mitralign Inc.).

## Procedural Planning and Echocardiographic Evaluation for Patient Selection

Patients referred to transcatheter mitral valve repair are those whose high surgical risk represents a main contraindication for open-heart valve treatment. The exception to this standard may be considered when a concomitant coronary artery disease requires adjuvant revascularization. When a patient is deemed inoperable, anatomical factors should carefully be considered for the selection of the appropriate intervention. Echocardiography, mostly TEE, is decisive for this aim.

### Echocardiographic Evaluation of FMR

**Figure 5:**
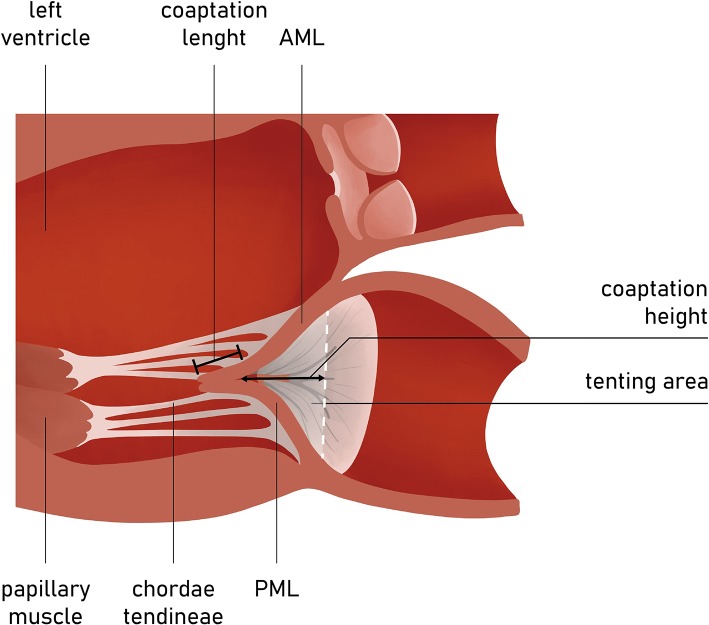
Long-axis view of the LV and MV apparatus. Coaptation length, height, and tenting area are among key measurements in the preoperative planning and screening.

The FMR is typically characterized by the unaltered leaflet structure; however, one should expect other abnormalities (e.g., ruptured chords, flail leaflet) that would imply primary pathology. In addition, quantitative assessment of some mitral apparatus structures facilitates the understanding of the valve disease etiology and may influence future screening and procedural planning (Figure 5).

The presence of leaflet apical tethering resulting in the apically displacement of coaptation point is a decisive echocardiographic feature of FMR. Noteworthy, the leaflet tethering forces cause impaired leaflet coaptation within the annular plane, resulting in incomplete closure. Carpentier's classification of dysfunction is based on the opening and closing motions of the mitral leaflets in relation to the annular plane. The FMR can be defined according to Carpentier mechanisms as type I and type III (11). Type I FMR is less frequently causing severe MR and it is related to annular dilatation with normal leaflet motion. Isolated LA dilation, without LV enlargement or dysfunction, was previously demonstrated to be not sufficient for determining significant FMR (12). On the contrary, a recent study revealed that mitral leaflet area (MLA) adaptation, which occurs as a compensatory mechanism among patients presented with atrial fibrillation (AF) and isolated annular dilatation without LV dysfunction, becomes insufficient with greater annular dilatation (13, 14). The prevalence of type I FMR is growing due to aging of the population, increasing prevalence of concomitant long-standing persistent AF, and impaired LV diastolic function. In non-ischemic MR, usually the tethering is symmetric and the jet is central (Figure 5).

**Figure 6:**
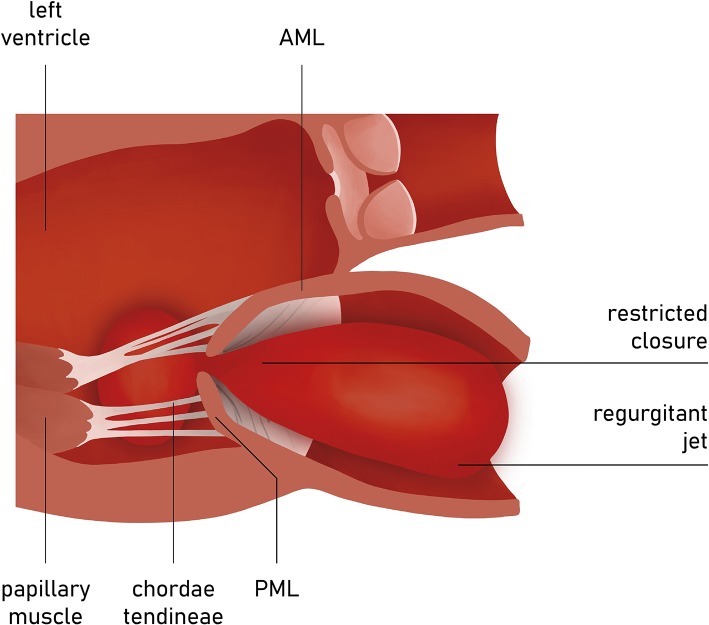
Carpentier type I mitral regurgitation (depending from annular dilatation). Normal leaflet motion. Regurgitation jet directed centrally.

**Figure 7:**
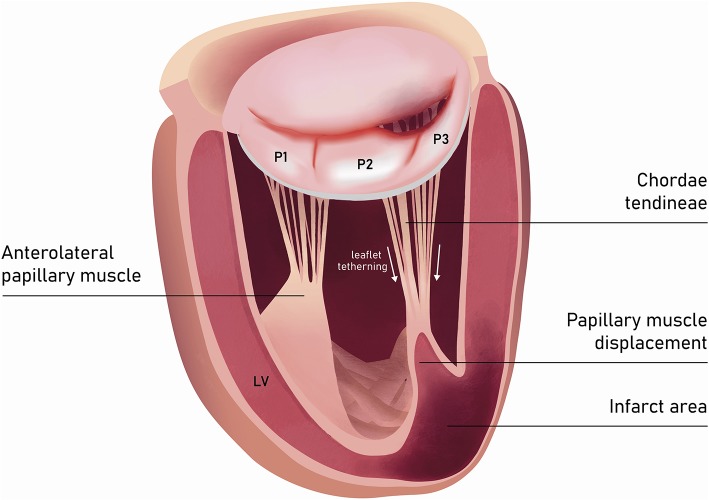
Left ventricle and mitral valve—side view (LVOT was not depicted). The restricted leaflet motion and tethering in the course of posterior papillary muscle ischemia predominantly affects the posteromedial leaflet segment (P3).

In the incidence of ischemic FMR (type IIIb), the displacement of the papillary muscles and distortion of LV directly alter the geometry and function of the mitral valve apparatus. Although both papillary muscles are frequently affected, it is the dysfunction of posterior papillary muscle that prevails and results in the tethering of the posteromedial leaflet segment (P3) (Figure 6). Hence, FMR jet is usually eccentric and directed posteriorly along the P3 area (Figure 7) (15). However, in the case of unpopular LAD infarction, the large central MR jets mark the preceding broad ischemia of the papillary muscles and other affected LV segments (16). With the increase of lateral and apical forces, the leaflet tethering predominates over annulus dilatation. In that case, the annuloplasty as a stand-alone procedure is typically not a sufficient repair therapy. Numerous surgical methods have been introduced to address the left ventricle dilatation (e.g., papillary muscle approximation, ventricular containment/ventriculoplasty). Nonetheless, having in mind further LV remodeling in the course of the secondary MR, the “downsizing” of the mitral annuloplasty (i.e., restrictive mitral ring implantation) may be recommended (14, 16, 17).

### Patient Selection and Success Predictors of Annuloplasty Techniques

Echocardiographic assessment should consistently clarify if the mechanism of MR is functional and at the same time evaluate the LV dimension and function to distinguish type I from type III FMR. Once the MR mechanism is determined, further TEE assessment is crucial for selecting the suitable patient for the right procedure (see section Anatomical Factors: MV Leaflet Tethering).

Presuming that MR is successfully treated with MitraClip, given the large amount of evidence, transcatheter edge-to-edge treatment may be regarded as a first-line procedure. Nonetheless, in the case of annular dilatation, one should consider annuloplasty techniques. In fact, this procedure “leaves the door opened” to other supplementary treatment (18, 19).

Evaluation of remodeling of MV apparatus and LV can facilitate the prediction of the MR recurrence after annuloplasty. From an anatomical perspective, the rationale of both surgical and transcatheter annuloplasty depends on shifting the posterior annulus anteriorly, reducing the septolateral distance and increasing the coaptation area. However, this mechanism does not settle anteriorly the coaptation point, since the posterior leaflet struggles being tethered posteriorly and its anterior excursion is noticeably limited. This phenomenon of “freezing of posterior leaflet” is always present after annuloplasty and does not affect the anterior leaflet (AL) motion, so that valve closure becomes essentially a “single-leaflet process” and the frozen posterior leaflet (PL) serves only as a support for the closure (16).

#### Anatomical Factors: MV Leaflet Tethering

Some MV geometry parameters have been found to be independent predictors of the recurrence of MR after MV ring annuloplasty. Previous studies have demonstrated that poor outcomes of the surgical annuloplasty may be associated with greater preoperative leaflet tethering. Calafiore et al. have determined that a coaptation distance of more than 1.1 cm might be associated with a high risk of MR recurrence after surgery (17). Likewise, a similar study of patients who underwent ring annuloplasty revealed that one should consider a posterior leaflet angle of more than 45°, a tenting area above 2.5 cm^2^, and the coaptation distance of more than 1 cm to predict the persistence of more than moderate MR after surgical annuloplasty (19). Considering the small amount of data of new-generation annuloplasty techniques, these parameters might also serve as a reference for transcatether MV repair (19–21).

**Figure 8:**
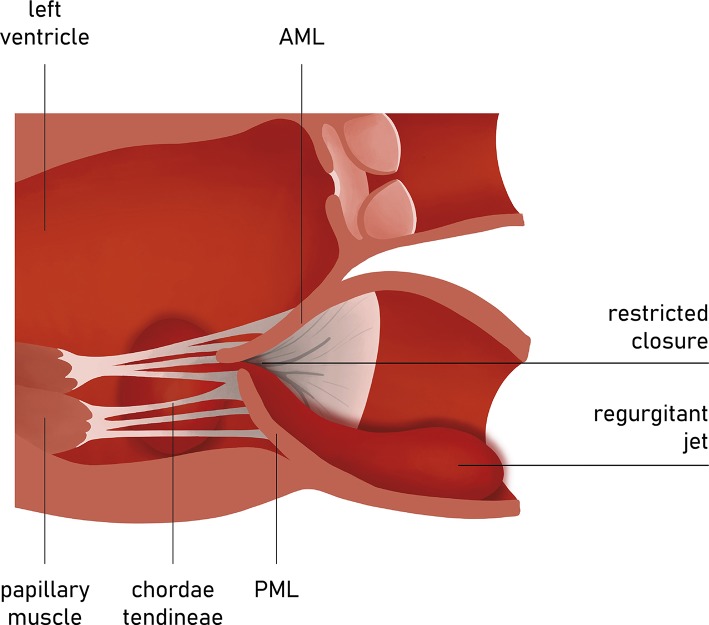
Carpentier type III mitral regurgitation. Restricted leaflet motion. Regurgitation jet directed eccentrically.

A tethering direction has been regarded as a determinant for the symmetry of the leaflet restriction. The properly functioning chordae system comprises the marginal part that fixes the free edges of both mitral leaflets and the stiffened basal part that constitute the base of the ventricular side of AL. Anterior leaflet tenting angles can be identified as basal and apical (Figure 8). Since the insertion points of chordae vary, the direction of tethering forces creates different geometrical shapes of tenting area and leaflets. When the posterior tethering predominates, the tethering of the basal chordae on the medial part of AL is more pronounced than the tethering of distal-primary chordae on the anterior leaflet tips. As a result, the AL structure tends to bend. This effect might be restored by reducing the posterior dilatation and the tethering on basal chordae. However, in the incidence of the apical tethering of both leaflets, the motion of distal AL is usually restricted, creating more challenging conditions for the successful annuloplasty (22). Hence, jet eccentricity, direction, and distal anterior leaflet tenting angle are currently regarded as the determinants for predicting the success of annuloplasty techniques, more than a basal anterior leaflet tenting angle and posterior tenting angle (23, 24). Annular size and calcifications as well as the proximity of the circumflex artery are also acknowledged factors of the feasible and successful direct annuloplasty.

#### Functional Factors: Left Ventricle Remodeling

Many studies, including more than 700 patients treated with MV annuloplasty, sought to identify the factors that correlate with mitral annuloplasty outcomes. In one analysis, the investigators revealed that the recurrence of MR within the first 6 months was associated with higher grade of preoperative MR, smaller body size, early date of operation, jet direction other than posterior (essentially central or complex), and the Peri-Guard annuloplasty technique (25). Subsequent MR recurrence (>6 months) was related to severe preoperative left ventricular dysfunction (26). More recently, a re-analysis of 214 patients with ischemic FMR by the Cardiothoracic Surgical Trials Network (CTSN) investigators, has demonstrated that the 1-year recurrence of MR is linked to LV end-systolic diameter/ring size mismatch, after adjustment for age, sex, and baseline LVEF. A basal aneurism or dyskinesis of LV was associated with significant recurrent MR, rebating the role of higher LV–MV ring mismatch that is obviously more pronounced in case of basal aneurysms (26, 27). In a small group of patients undergoing MV ring annuloplasty, it was demonstrated that the LV sphericity index, calculated at end-systole as the volume of LV divided by the volume of a sphere with a diameter equal to the LV longest axis (measured in apical view), was the best predictor of long-term recurrent MR. Indeed, this parameter conveys the degree of tethering, as the more spherical is the LV, the more the displacement of the papillary muscle (25). Keeping in mind that advanced LV remodeling is associated with worse outcomes, in the subgroup of patients with enlarged ventricle and great tenting height (particularly >11 mm), not only the annuloplasty, but a subvalvular repair might be necessary (28).

It has to be noted that all these factors (anatomical and functional) derive from surgical setting and further studies are necessary to prove them as predictors of the recurrence of MR after transcatheter annuloplasty. Despite the lack of evidence, these criteria can be useful in preoperative evaluation of patients candidate to transcatheter annuloplasty for predicting the risk of procedural failure. Noteworthy, in the intraprocedural setting of transcatheter intervention, these factors can be re-evaluated right after the restrictive annuloplasty in order to plan further staged or even combined interventions (Table 2) (17, 18).

**Table 2:** Predictors of failure of annuloplasty evaluated in surgical setting.

**Predictors of recurrent MR after surgical annuloplasty**
**Short-term results**
Distal anterior leaflet angle tethering
Leaflet tethering: tenting depth (height) and tenting area
Higher grade of preoperative MR
Smaller body size
Early date of operation
Jet direction other than posterior (essentially central or complex)
Peri-Guard annuloplasty technique
**Mid- and long-term results**
Severe LV dilatation and dysfunction
LV sphericity
Ratio between LVESD/ring size (mismatch)
Basal aneurysm/dyskinesis
Clinical factors/past history (age, body mass index, sex, race, NYHA, prior CABG, prior percutaneous coronary intervention, and history of ventricular arrhythmia)

## Coronary Angiography and Computed Tomography (CT)

**Figure 9:**
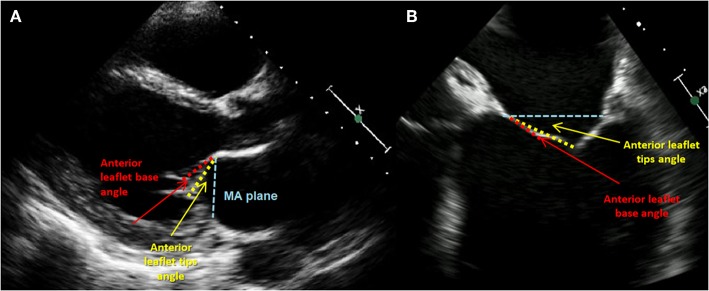
Phenotypes of tethering. **(A)** Transthoracic echocardiography (TTE), parasternal LAX view: anterior leaflet (AL) tethering with AL bend; **(B)** transesophageal echocardiography (TTE), midesophageal 4-CH view focused on MV: less pronounced bend and tip angle of the AL.

The presence and significance of possible coexisting coronary artery disease need to be documented; therefore, coronary angiography is routinely performed. Over the years, together with a rapid development of percutaneous interventions, computed tomography imaging has become a decisive tool for the preprocedural planning and implant selection. CT reconstructions allow a comprehensive assessment of mitral valve anatomy and can be a valuable tool to predict procedural challenges such as the proximity of the adjacent structures, in particular the circumflex artery and the risk of its injury (29, 30). Along with the MV annular size and aortic and mitral valve correlations, the optimal transseptal puncture site is selected. Secondly, the posterior annulus is divided into multiple regions to identify the right position and indicate the anchor angle. Finally, optimal fluoroscopic planes for implantation should be estimated. Keep in mind that mitral annular calcification might be considered as a contraindication for mitral annuloplasty, as severe MAC hinders optimal anchoring and contracting of the implant and may also deteriorate echocardiographic image quality (Figure 9).

## Cardioband Mitral System (Edwards Lifesciences)

The early experience with the Cardioband proved that this direct annuloplasty device is feasible, safe, and effective. The implantation is performed under general anesthesia with fluoroscopy and 3D-echo guidance in a stepwise fashion. While proceeding with the procedure, every move is reversible, resulting in a legitimate safety level and control.

**Figure 10:**
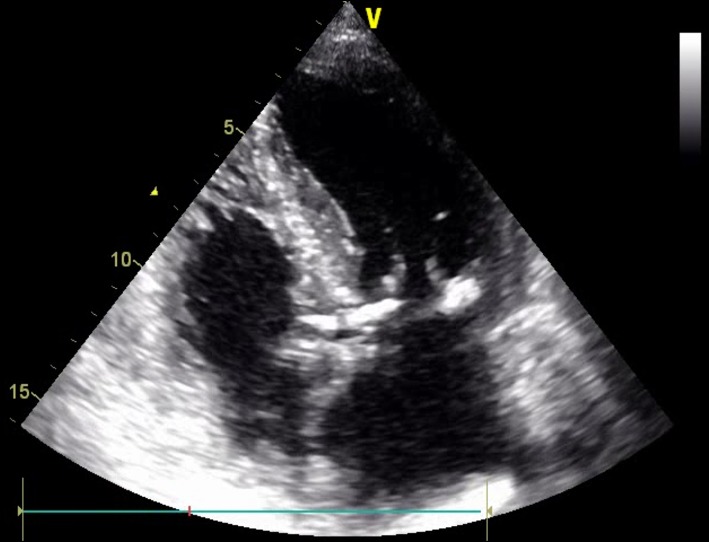
Transthoracic echocardiography (TTE); apical 4-CH view. Mitral annular calcification (MAC).

**Figure 11:**
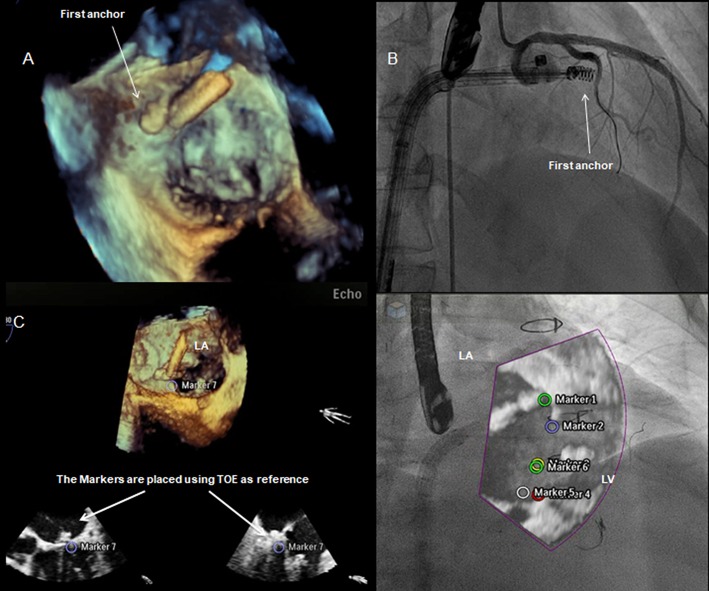
Integrated intra-procedural guidance for Cardioband: **(A,C)** real-time 3D transesophageal echocardiography facilitates the navigation and delivery of the first anchor; **(B)** fluoroscopy: the use of coronary wire may serve as a radiographic marker and a potential railway for bailout strategies. Fusion imaging allows to display an overlap image of the “echo-structures” in the fluoro-images. This could be a valuable tool in the presence of difficult anatomies and in the first phase of the operator's learning curve.

**Figure 12:**
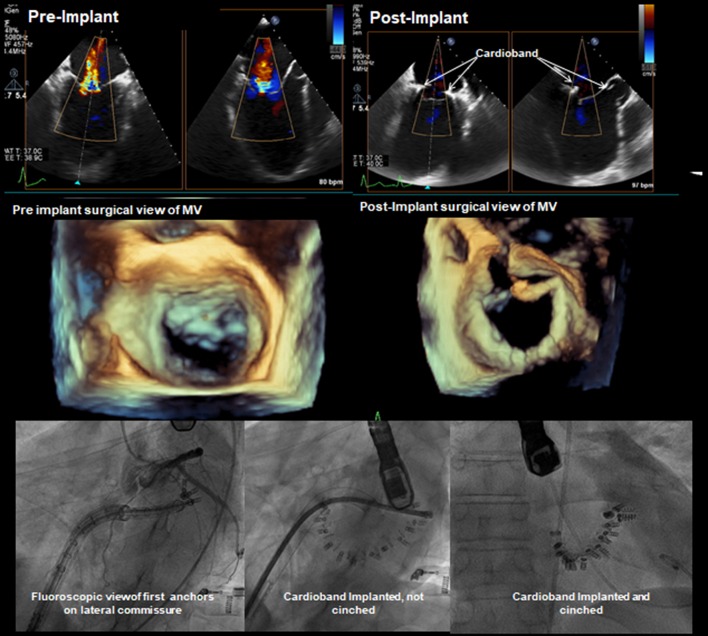
Pre- and post-implantation echo and fluoroscopy images. TEE 2-CH and LAX projections demonstrating visibly reduced regurgitation jet. Fluoro images showing the process of deployment and clinching of the annuloplasty ring concluded with a successful implantation.

The Cardioband system uses a transseptal steerable sheath (TSS) that is delivered over a super-stiff guide wire via the femoral vein into the left atrium by a standard transseptal puncture. By that time, make sure to obtain an activated clotting time between 250 and 300 s. The steerable sheath facilitates the optimal positioning of the implant catheter possibly close to the leaflet hinge, near the anterior commissure. Verification of the first anchoring location is crucial and requires echo supervision to prevent the damage of surrounding structures. To rule out the risk of circumflex artery injury, coronary angiography is performed. The use of a standard 0.014″ coronary wire may serve as a radiographic marker and a potential railway for bailout PCI-LCx. Regardless of the assessment, the operator needs to be prepared for a possible bail-out scenario (Figure 10) (31). The depicted vessel might also serve as a useful reference to guide the procedure. After the first implant is delivered at the anterolateral trigone and its position is verified, a set of anchors is advanced through the polyester sleeve into the annular part in the posterior and medial correspondence to the first implant. Every anchor is deployed until the radiopaque marker on the Implant Catheter Channel reaches the next marker on the implant. Before every release, the operator needs to verify proper anchoring of the implant with “push-and-pull test” under echo and fluoroscopic guidance. Please note that the implantation of the anchors with an angle of 45° can improve the fixation permanence. The number of anchors depends on the size of the device implanted and are usually delivered every 8 mm until the Implant Catheter tip reaches the final anchoring position on the posterior commissure. Usually, during the anchor implantation into the myocardial tissue, extra-systolic beats on the ECG can be observed. When the final anchor is deployed, the implant is detached from the delivery system and removed. Once the entire device is implanted, the contraction wire with “size adjustment tool” is advanced over the implant guide wire, until the distal tip reaches the “adjustment spool” of the implant. The implant is then contracted by clockwise turn of the “adjustment roller” until the appropriate size is reached. Typically, a left anterior oblique (LAO) projection is used to guide this step. Finally, the obtained reduction of MR is evaluated by TEE under beating-heart setting (Figures 11, 12).

The key advantages of the system are adjustable implantation with the real-time confirmation of the result due to echo-driven live imaging and the instantaneous improvement of the patient's hemodynamics. The device preserves the patient's native anatomy, keeping future treatment options open in case of recurrent MR. The recent studies proved the efficacy and safety of the Cardioband implantation, which resulted in a significant MR reduction in majority of patients and was associated with significant improvement in functional status and quality of life (32). The ongoing ACTIVE randomized trial is expected to support these early promising results (NCT03016975).

## The Mitralign (Mitralign INC.)

The Mitralign uses a retrograde approach to deliver a 14-Fr guiding catheter through the aortic valve, onto the posterior side of the left ventricle, beneath the mitral leaflet. Two pairs of wires are advanced through the MA tissue and deliver the pledges on both sides of the commissure. The first pledged catheter extrudes half the pledget on the atrial side and the other on the ventricular side. The delivery of the second pledged catheter follows the same steps. Both anchors are connected by a drawstring, and by tightening up the sutures, the reduction of MA is achieved. When the same steps are followed on the opposite side of the valve, the procedure is complete. With the Mitralign System, the perimeter of the annulus can be reduced by as high as 20%. The device is assigned to FMR patients, addressing MV annular dilatation. The key advantage of this technique is that the transseptal puncture is not needed during the procedure. However, in patients with depressed LV function, the retrograde access may be less tolerated. In the “Mitralign Percutaneous Annuloplasty First-in-Man Study,” the device proved the favorable safety profile while reducing the MR grade and symptoms in 50% of patients during 6-month observation (33).

## Millipede Transcatheter Annuloplasty Ring (Boston Scientific Corp.)

The direct transcatheter-based annuloplasty approach from Millipede features a complete semi-rigid ring designed to reproduce the surgical MV repair. The delivery system includes the guide catheter, the delivery catheter, and the ICE catheter. Introduced by transfemoral, the venous approach is delivered through the transeptal puncture above the mitral valve annulus. The delivery catheter settles the device supra-annularly just before the anchoring. Finally, the ring is clinched, resulting in the annular size reduction (anterior–posterior diameter). The mechanism is designed to provide reposition and retrieve options during the procedure, while preserving the possibility for further sub-valvular treatment after complete implantation. The procedure is guided by the compound, but standard imaging: fluoroscopy to assess the atrium, TEE to land the device, and ICE to locate the anchors. The introduced steering method facilitated by the ICE imaging might be particularly convenient for experienced MitraClip implanters. To date, the device proved encouraging safety profile and efficacy in reducing MR. The technology is still under development and not available for commercial use (34).

## Conclusions

Patients with severe MR regarded too high risk for surgery can benefit from novel percutaneous approaches. Current imaging modalities have contributed to understanding the etiology and anatomy of MR, and are an integral part of preprocedural planning. Considering that TEE and CT offer a comprehensive 3D assessment, they are regarded as the gold standard for MR evaluation and the optimal patient selection. The enhanced live visualization of the mitral apparatus due to the fusion of 3D-echo and fluoroscopy can provide more intuitive imaging and facilitate the intraprocedural guidance at any stages of the annuloplasty procedure (33).

The direct percutaneous mitral annuloplasty addresses the underlying mechanisms of FMR with a less invasive, catheter-delivered approach. In previously conducted studies, based on surgical experience, the investigators demonstrated that the combination of leaflet repair with surgical annuloplasty has a potential for a lower rate of MR recurrence (35). Although the percutaneous annuloplasty devices are primarily intended for the stand-alone treatment of FMR, the simultaneous usage of two therapies may become an alternative strategy for some subgroups (36). Therefore, Latib et al. proved the feasibility of percutaneous direct annuloplasty as a treatment option for patients with FMR previously subjected to MitraClip and presented with persistent annular dilation and recurrent MR. As in surgical setting, the direct transcatheter annuloplasty may serve as a part of the combination strategy with a percutaneous edge-to-edge repair for individuals with a functional impairment of mitral valve and asymmetric tethering to obtain lower rates of MR recurrence. Yet, this has to be proven in further clinical trials. Moreover, some patients screened for MitraClip are considered unsuitable, indicating that there is a clinical need for an adjunctive transcatheter mitral repair strategy (37–39). This group primarily includes individuals with calcified or rheumatic deformed leaflets and substantial annular dilatation. Although there has not been any cutoff for the annular dimension established, one may consider a range between 40 and 45 mm too much for the edge-to-edge therapy as a first-line procedure. However, in some cases, despite the extensively dilated annulus, the length of the leaflets may still allow sufficient coaptation. One should consider isolated annuloplasty procedure for an early treatment of the FMR, while the ideal patient for that indication might be regarded as the one with limited tethering of the posterior leaflet or with the isolated atrial enlargement with a concomitant annular dilatation (in the absence with LV remodeling).

The recently published propensity matched analysis of registry data demonstrated that both edge-to-edge treatment and direct annuloplasty effectively reduce MR and heart failure symptoms. However, FMR patients treated with Cardioband proclaimed more notable improvement regarding NYHA scale, rehospitalization, and mortality, with predominant benefit in the EF < 30% subgroup (40).

The here-described devices proved to be safe and effective, providing significant MR reduction in the study group and significant improvement of symptoms (30, 31). Finally, the simultaneous training of interventional cardiologists, cardiosurgeons, and echocardiographers, and reproducibility of the procedure are the key points to achieve more optimal results with less procedural times. Having in mind all mitral therapeutic options, current cardiovascular medicine offers a therapy based on the mechanism of MR with new approaches likely to set a new standard of treatment in the forthcoming future.

## Author Contributions

TG: literature selection, personal experience, and main contribution in paper writing. MG: literature selection, personal experience, and second-main contribution in paper writing. MT: paper structure conception, paper writing guidance, and practical experience. MZ and FM: medical consultation and practical experience.

### Conflict of Interest

MT: consultant for Abbott Vascular, Boston Scientific, 4tech, CoreMedic. Speaker fees from Edwards Lifesciences. FM: consultant Abbott, Edwards Lifesciences, Medtronic, Perifect, Transseptal solutions, Xeltis, Cardiovalve; Grant receiver: Abbott, Medtronic, Edwards Lifesciences, Biotronik, Boston Scientific; Royalties receiver: Edwards Lifesciences, 4 Tech. MG: consultant for Biotronik. The remaining authors declare that the research was conducted in the absence of any commercial or financial relationships that could be construed as a potential conflict of interest.

## Abbreviations

MR: mitral regurgitation
FMR: functional mitral regurgitation
HF: heart failure
LV: left ventricle
MAC: mitral annular calcification
CT: computed tomography
TEE: transesophageal echocardiography
MA: mitral annulus
LV: left ventricle
LAD: left anterior descending (artery)
LA: left atrium
AF: atrial fibrillation
PL: posterior leaflet
AL: anterior leaflet
CABG: coronary artery bypass grafting
ICE: intracardiac echocardiography.
